# Impact of antimicrobial resistance genes and phenotypic susceptibility profiles on growth fitness in nontyphoidal *Salmonella enterica* from U.S. retail meat surveillance

**DOI:** 10.1016/j.onehlt.2026.101642

**Published:** 2026-10-30

**Authors:** Maria Camila Duarte-Villanueva, Gregory H. Tyson, Tara N. Gaire

**Affiliations:** a Texas Tech University School of Veterinary Medicine, Amarillo, TX 79106, USA; b U.S. Food and Drug Administration, Laurel, MD, 20910, USA

**Keywords:** *Nontyphoidal Salmonella enterica*, Antimicrobial resistance, Growth fitness, Retail meat surveillance, Multidrug resistance

## Abstract

Nontyphoidal *Salmonella enterica* subsp. *enterica* (*S. enterica*) is a major foodborne pathogen, and reduced susceptibility or resistance to ceftriaxone (cephalosporins) and ciprofloxacin (fluoroquinolone) is a public health concern because these drugs are important treatment options for severe salmonellosis. Acquisition of antimicrobial resistance can affect the adaptation and evolution of *Salmonella* and is often assumed to impose a biological fitness cost, which may reduce the ability of resistant strains to persist in the absence of antimicrobial selection pressure. However, whether contemporary retail meat associated *S. enterica* strains with clinically relevant resistance phenotypes or genotypes exhibit reduced growth fitness remains unclear. Using longitudinal optical density–based growth assays with a standardized bacterial starting density, we evaluated the in vitro growth fitness of twenty-five nontyphoidal *S. enterica* subsp. *enterica* strains collected through the U.S. FDA National Antimicrobial Resistance Monitoring System (NARMS) retail meat surveillance program. The strain set included diverse serovars, different levels of susceptibility to ceftriaxone and ciprofloxacin, β-lactam and fluoroquinolone resistance mechanisms, and multidrug resistance profiles. Mathematical growth models were used to estimate intrinsic growth rate, lag duration, and maximum stationary-phase density under nonselective conditions. Overall, strains with reduced susceptibility or resistance to ceftriaxone or ciprofloxacin did not consistently exhibit reduced intrinsic growth rates compared with susceptible strains. Strains carrying β-lactam resistance genes, including *bla*_CMY-2_, *bla*_CTX-M-65_, or fluoroquinolone resistance mechanisms, including *qnrB19* and the *gyrA* D87Y mutation, generally maintained growth fitness comparable to strains lacking these AMR genes. Similarly, multidrug-resistant *S. enterica* serovars retained comparable in vitro growth fitness despite carrying diverse resistance genes across antimicrobial classes. These findings suggest that clinically relevant antimicrobial resistance in retail meat–associated *S. enterica* is not necessarily accompanied by reduced in vitro growth fitness. The absence of a consistent growth disadvantage may facilitate persistence of resistant *Salmonella* in food-associated bacterial populations even when antimicrobial selection pressure is reduced, underscoring the need for integrated genomic, phenotypic, and One Health surveillance.

## Highlights

• Retail meat–associated *Salmonella enterica* with clinically relevant AMR retained comparable in vitro growth fitness.
• Ceftriaxone and ciprofloxacin resistance phenotypes were not consistently linked to reduced intrinsic growth rate.
• β-lactam and fluoroquinolone resistance mechanisms did not impose a consistent measurable growth disadvantage.
• Comparable growth fitness may support persistence of resistant *Salmonella* in food-associated bacterial populations.

## 1 Introduction

Foodborne diseases caused by nontyphoidal *Salmonella enterica* subsp. *enterica* (*S. enterica*) represent a major public health problem in the United States and worldwide [1]. Most human infections result from the consumption of contaminated foods of animal origin, including poultry, beef, pork, and eggs. While most cases are self-limiting, severe or invasive infections require antimicrobial therapy. In such cases, ciprofloxacin (a fluoroquinolone) and ceftriaxone (a third-generation cephalosporin) are critical treatment options [2]. The emergence and persistence of antimicrobial resistant (AMR) *Salmonella* associated with these clinically important antimicrobials is therefore a significant food safety and public health concern [3], [4].

However, the long-term public-health impact of this resistance depends in part on the microbial fitness of resistant *S. enterica* strains—that is, whether they retain the ability to grow and compete relative to susceptible strains [5]. Antimicrobial resistance is often assumed to impose a biological fitness cost on bacteria [6], typically inferred from growth rate [7], lag phase, or maximum population density [8] [9]. This fitness cost is thought to be largely due to the resources bacteria must devote to maintaining plasmids and producing resistance-associated proteins that provide no benefit in the absence of selective pressure [10]. In the context of foodborne pathogens such as nontyphoidal *S. enterica*, the presence or absence of a growth-fitness cost (or advantage) has implications for AMR surveillance. For example, resistant *Salmonella* strains that maintain a growth advantage may persist and disseminate effectively through food-production systems and retail meat, even when antimicrobial selective pressure decreases. Consequently, the magnitude of any fitness effect can influence the likelihood that resistance will remain stable within bacterial populations. A few studies documented the effect of resistance to clinically relevant antimicrobials on the fitness of *Salmonella* [11], [12], [13]*.* However, it remains unclear whether contemporary U.S. retail meat–associated *S. enterica* strains resistant to ceftriaxone or ciprofloxacin, or those encoding β-lactam or fluoroquinolone resistance mechanisms, exhibit measurable reductions in growth fitness across diverse nontyphoidal *Salmonella* serovars, antimicrobial classes, and AMR genes.

Therefore, we designed the present study to determine whether nontyphoidal *S. enterica* strains less susceptible or resistant to ceftriaxone and ciprofloxacin carry a range of clinically and epidemiologically relevant AMR genotypes and phenotypes that could influence growth rates. These isolates were collected through the U.S. Food and Drug Administration (FDA) *National Antimicrobial Resistance Monitoring System* (NARMS) retail meat surveillance program and represent diverse serovars and multidrug-resistance phenotypes (MDR; resistance to ≥3 different antimicrobial classes) [14]. We hypothesized that *S. enterica* strains with higher ceftriaxone or ciprofloxacin minimum inhibitory concentrations (MICs), or those carrying β-lactam or fluoroquinolone resistance mechanisms, would exhibit reduced in vitro growth fitness compared with susceptible isolates. To test this hypothesis, we performed in vitro growth-curve assays and fitted mathematical growth models to quantify intrinsic growth rate, lag-phase duration, and stationary-phase density across clinically and epidemiologically relevant *S. enterica* isolates from retail meats.

Here, we show that growth fitness varied across retail meat–associated nontyphoidal *S. enterica* strains by ceftriaxone- and ciprofloxacin-associated genotypes, serovars, and multidrug-resistance profiles, but resistant genotypes and phenotypes did not consistently show reduced fitness compared with susceptible strains. This suggests that resistant strains may maintain growth fitness comparable to that of susceptible strains under the tested in vitro conditions. These findings have important One Health implications and underscore the importance of routine surveillance for MDR *Salmonella* serovars.

## 2 Materials and methods

### 2.1 Salmonella strains

A total of twenty five nontyphoidal *S. enterica* strains were used in this study. The strains were obtained from the FDA NARMS retail meat surveillance program, where state public health labs and departments of agriculture, as well as universities, conduct routine sampling of meats and test for the presence of *Salmonella*, among other bacteria. The strains were isolated from retail meat products collected between 2019 and 2021 across multiple U.S. states, including Texas (*n* = 7), Minnesota (*n* = 5), California (*n* = 2), Colorado (*n* = 2), Connecticut (*n* = 2), Kansas (*n* = 2), New York (*n* = 2), Georgia (*n* = 1), Maryland (*n* = 1), and Washington (*n* = 1). The collection included isolates from retail chicken (*n* = 12), pork chops (*n* = 8), ground turkey (*n* = 4), and ground beef (*n* = 1) (Table S1). Strains were selected to represent diverse serovars, antimicrobial susceptibility profiles, and AMR genes relevant to ceftriaxone and ciprofloxacin. The study was designed to evaluate growth fitness among selected isolates rather than to estimate the prevalence of *Salmonella* in retail meat.

The strains represented diverse *S. enterica* serovars: Kentucky (*n* = 4), Infantis (*n* = 4), Typhimurium (*n* = 4), Johannesburg (*n* = 3), Derby (*n* = 2), Senftenberg (*n* = 2), and I 4, [5],12:i:- (*n* = 3), with one strain each of Alachua, Braenderup, and Reading (Table 1; Table S1). Serovar assignments were obtained from the NARMS isolate characterization records and associated genomic surveillance data.

**Table 1:** Characteristics of the 25 *S. enterica* isolates included in the study. Detailed isolate-level information, including retail meat sources, collection locations, antimicrobial susceptibility profiles, resistance genes, and whole-genome sequencing accession numbers, is provided in Supplementary Table S1.

Isolate No.	*S. enterica* Serotype	Source	Antibiotic Resistance Profile	β-lactam/FQ Resistance Mechanism Present	AMR Genes
1	Alachua	Ground turkey	Amc, Amp, Axo, Chl, Fis, Fox, Suf, Tet	Yes	aph(3″)-Ib, aph(3′)-Ia, aph(6)-Id, bla_CMY-2_, sul2, fosA7.8, bla_TEM-1_, cmlA5, floR, tet(A)
2	Braenderup	Pork chops	Amc, Amp, Axo, Chl, Fis, Fox, Gen, Suf, Tet	Yes	ant(2″)-Ia, aph(3″)-Ib, aph(3′)-Ia, aph(6)-Id, bla_CMY-2_, sul2, bla_TEM-1_, cmlA5, floR, tet(A)
3	Derby	Pork chops	Cip, Nal, Tet	Yes	fosA7.3, qnrB19, tet(B)
4	Derby	Pork chops	Cip, Fis, Suf, Tet	Yes	aadA2, sul1, fosA7.3, qnrB19, tet(A)
5	I 4, [5],12:i:-	Chicken	Amp, Cip, Nal, Tet	Yes	bla_TEM-1_, qnrB19, tet(B)
6	I 4, [5],12:i:-	Ground beef	Amp, Cip, Fis, Nal, Suf, Tet	Yes	aph(3″)-Ib, aph(6)-Id, sul2, bla_TEM-1_, qnrB19, tet(B)
7	I 4, [5],12:i:-	Ground turkey	Amp, Cip, Nal, Tet	Yes	bla_TEM-1_, qnrB19, tet(B)
8	Infantis	Chicken	Amp, Axo, Chl, Cip, Cot, Fis, Gen, Nal, Suf, Tet	Yes	aac(3)-IVa, aadA1, aph(3′)-Ia, bla_CTX-M-65_, dfrA14, sul1, floR, gyrA D87Y, tet(A)
9	Infantis	Chicken	Amp, Axo, Chl, Cip, Cot, Fis, Gen, Nal, Suf, Tet	Yes	aac(3)-IVa, aadA1, aph(3′)-Ia, bla_CTX-M-65_, dfrA14, sul1, floR, gyrA D87Y, tet(A)
10	Infantis	Chicken	Amp, Axo, Chl, Cip, Fis, Gen, Nal, Suf, Tet	Yes	aadA1, bla_CTX-M-65_, sul1, bla_TEM-1_, gyrA D87Y, tet(A)
11	Infantis	Ground turkey	Amp, Axo, Cip, Fis, Nal, Suf, Tet	Yes	aadA1, bla_CTX-M-65_, sul1, blaTEM-1, gyrA D87Y, tet(A)
12	Johannesburg	Pork chops	Cip	Yes	qnrB19
13	Johannesburg	Pork chops	Amp, Chl, Cip, Fis, Gen, Nal, Suf, Tet	Yes	aac(3)-IVa, aadA7, sul1, bla_TEM-1_, qnrB19, tet(B)
14	Johannesburg	Pork chops	Amp, Chl, Cip, Fis, Gen, Nal, Suf, Tet	Yes	aac(3)-IVa, aadA7, sul1, bla_TEM-1_, qnrB19, tet(B)
15	Kentucky	Chicken	None	No	aph(3″)-Ib, aph(6)-Id
16	Kentucky	Chicken	Tet	No	aph(3″)-Ib, aph(6)-Id, tet(B)
17	Kentucky	Chicken	None	No	aph(3″)-Ib, aph(6)-Id
18	Kentucky	Chicken	Amc, Amp, Axo, Fox	Yes	bla_CMY-2_
19	Reading	Ground turkey	Amc, Amp, Cip, Fis, Nal, Suf,Tet	Yes	aph(3″)-Ib, aph(6)-Id, sul2, bla_TEM-1_, qnrB19, tet(A)
20	Senftenberg	Ground turkey	Amc, Amp, Axo, Fox	Yes	blaCMY-2
21	Senftenberg	Pork chops	Cip, Nal	Yes	qnrB19
22	Typhimurium	Chicken	Amc, Amp, Axo, Fis, Fox, Suf, Tet	Yes	bla_CMY-2_, sul2, tet(A)
23	Typhimurium	Chicken	Amc, Amp, Axo, Fis, Fox, Suf,Tet	Yes	bla_CMY-2_, sul2, tet(A)
24	Typhimurium	Chicken	Amc, Amp, Axo, Fis, Fox, Suf, Tet	Yes	bla_CMY-2_, sul2, bla_TEM-1_, tet(A)
25	Typhimurium	Chicken	Fis, Suf, Tet	No	sul2, tet(A), tet(C)

Amc, amoxicillin–clavulanate; Amp, ampicillin; Axo, ceftriaxone; Chl, chloramphenicol; Cip, ciprofloxacin; Cot, trimethoprim–sulfamethoxazole; Fis, sulfisoxazole; Fox, cefoxitin; Gen, gentamicin; Nal, nalidixic acid; Suf, sulfamethoxazole; Tet, tetracycline; FQ fluoroquinolone; None, no resistance detected.

The FDA NARMS routinely collects whole-genome sequencing (WGS) data for *Salmonella* isolates to characterize AMR genes and resistance-associated mutations. For the isolates included in this study, WGS data were generated by participating NARMS retail meat laboratories using Illumina sequencing platforms following established protocols and quality control procedures. The resulting raw sequencing reads were deposited in the National Center for Biotechnology Information (NCBI) Sequence Read Archive (SRA) and processed through the NCBI Pathogen Detection pipeline. Serovar information was obtained from NARMS isolate characterization records, which use SeqSero for computed serotypes [15], while AMR genes and resistance-associated chromosomal mutations were identified using AMRFinderPlus [16]. The corresponding SRA accession numbers are provided in Supplementary Table S1.

For each strain, phenotypic susceptibility MICs were determined by broth microdilution according to Clinical and Laboratory Standards Institute (CLSI) methods [17] (Table S1). The ceftriaxone study included strains without *bla* genes and strains harboring beta-lactamase genes of various spectra, including *bla*_*CMY-2*_, *bla*_*CTX-M-65*_, and *bla*_*TEM-1*_, with phenotypic susceptibility (MIC range: ≤0.25 to 64 μg/mL). Likewise, the ciprofloxacin study included strains with and without fluoroquinolone resistance mechanisms, including *qnr*B and the gyrA D87Y mutation, with phenotypic susceptibility (MIC range: ≤0.015 to 1 μg/mL). These strains also carried genes encoding reduced susceptibility or resistance across multiple antimicrobial classes (Table S1).

Initial MIC determination was performed by broth microdilution, which reports MICs in two-fold dilution increments. Because this standard approach can yield relatively wide MIC intervals for some antimicrobial bacterial strain combinations, a subset of strains was retested using gradient diffusion *E*-tests to obtain more precise MIC estimates for ceftriaxone and ciprofloxacin. Briefly, isolates were recovered from frozen stocks on blood agar plates and incubated overnight at 37 °C. Well-isolated colonies were suspended in sterile 0.85% saline and adjusted to a 0.5 McFarland standard. The standardized bacterial suspension was lawn-inoculated onto Mueller–Hinton agar plates using a sterile swab. Liofilchem™ MTS™ gradient diffusion strips for ceftriaxone (0.016–256 μg/mL) and ciprofloxacin (0.002–32 μg/mL) were then applied to the agar surface according to the manufacturer's instructions. Plates were incubated at 37 °C for 24 h (h), and MICs were recorded at the point where the inhibition ellipse intersected the strip scale.

### 2.2 Preparation of *Salmonella* cultures

All strains were maintained as frozen glycerol stocks at −80 °C. Before experimentation, strains were streaked onto blood agar plates to obtain well-isolated colonies and incubated aerobically at 37 °C for 18 h. Well isolated colonies were then used for culture preparation and growth-fitness assays. *Salmonella* cultures were prepared by inoculating 1–2 isolated colonies from each BA plate into a tube containing 10 mL of Brain Heart Infusion (BHI) (Remel, Lenexa, KS, United States) broth. Cultures were incubated aerobically at 37 °C with continuous shaking at 200 rpm for 18 h. After incubation, overnight cultures were removed from the shaker incubator and used to standardize the starting density (∼10^5^ CFU/mL). Cultures were subjected to a two-step dilution procedure. First, 57.5 μL of each overnight culture was transferred into 10 mL of fresh sterile BHI broth (1174 dilution) and incubated for 1 h at 37 °C with shaking at 200 rpm to promote active log-phase growth. Subsequently, 250 μL of each subculture was transferred into 9.75 mL of fresh sterile BHI broth (1,40 dilution), yielding a final volume of 10 mL per strain at a target starting density of approximately 10^5^ CFU/mL. We further confirmed this standardized starting bacterial density using bacterial counts (details below).

### 2.3 Growth curve assay

Growth kinetics were assessed using a 96-well flat-bottom plate (Costar, Washington, D.C., United States) in a on a Microplate Reader LX Multi-Mode Microplate Reader Incubator (Synergy LX Multi-Mode, BioTek, Winooski, VT, United States). Each of the 25 strains was assayed in biological triplicate (three independent wells), yielding 75 experimental wells. An additional 10 wells containing sterile BHI broth without bacteria were included as negative controls. *Escherichia coli* ATCC 1430, previously used in our laboratory as a control strain for antimicrobial susceptibility testing, was included in 10 additional wells as an internal growth control to monitor assay consistency across experimental runs and ensure that the growth curve assay was working as intended. This strain was not used as a biological reference for comparing growth fitness among *S. enterica* strains. Each well was loaded with 200 μL of the culture of standardized starting density (corresponding T_0_ culture), positive or negative control. The 96-well plate was sealed with a breathable adhesive sealing sheet (Thermo Fisher Scientific, Waltham, MA, United States) to allow gas exchange while minimizing evaporation, then placed in the plate reader incubator set to 37 °C with continuous orbital shaking. Bacterial growth was monitored by measuring optical density at 600 nm (OD_600_) every 30 min for 36 h to assure optimal visualization of all growth phases. OD_600_ values were background-corrected by subtracting the mean absorbance of the BHI control wells at each time point.

### 2.4 Bacterial enumeration by colony count

To check the initial and final bacterial density, viable plate counts were performed at T_0_ and at the end of the incubation period (0 and 24 h). At each time point, 100 μL aliquots were collected from each strain culture and subjected to serial 10-fold dilutions in sterile Buffered Peptone Water (BPW) (Remel, Lenexa, KS, United States). For Hour 0 samples, dilutions 10^−3^, 10^−4^, and 10^−5^ were plated; for Hour 24 samples, dilutions 10^−5^, 10^−6^, and 10^−7^ were plated, to account for the expected increase in bacterial density over the incubation period.

A 100 μL volume from each dilution was spread onto the surface of Tryptic Soy Agar (TSA) (Remel, Lenexa, KS, United States) plates by duplicate using sterile L-shaped spreaders. All TSA plates were incubated at 37 °C for 24 h. After the incubation time elapsed, colonies were enumerated. Only plates yielding 30–300 colonies were used for CFU/mL calculations. Bacterial density was expressed as CFU/mL using the formula: CFU/mL = colony count / (volume plated [mL] × dilution factor). A sterile BPW negative control was included in each plating session to confirm the absence of contamination.

### 2.5 Growth curve experiments and optical density processing

Salmonella growth dynamics were assessed using longitudinal optical density measurements. For each isolate, growth curves were generated in triplicate, and OD600 was measured over 0–36 h. Raw OD values were corrected using the corresponding blank wells, and corrected OD values were used for growth-curve modeling.

### 2.6 Mathematical modeling and prediction of in vitro growth

Longitudinal optical density (OD) data were analyzed using nonlinear growth-curve modeling. For each strain, OD was modeled as a function of time, t, in hours. Five candidate models were evaluated: 1) constant/no-growth model, 2) logistic model, 3) Gompertz model, 4) Richards model, and 5) Baranyi–Roberts model [7], [18], [19]. The constant model was defined as $\mathrm{OD}\left(t\right)=y_{0}$, where $y_{0}$represents the baseline OD. The logistic model was defined as [$\mathrm{OD}\left(t\right)=y_{0}+\frac{A}{1+\exp\left[-r\left(t-t_{\mathrm{mid}}\right)\right]}$], where Arepresents total OD increase, ris the logistic growth-rate parameter, and $t_{\mathrm{mid}}$is the inflection time. The Gompertz model was defined as [$\mathrm{OD}\left(t\right)=y_{0}+A\times \exp\left\{-\exp\left[\left(\mathrm{\mu e}/A\right)\left(\lambda -t\right)+1\right]\right\}]$, where μrepresents the maximum OD-based growth rate, λrepresents lag time, and eis Euler's number. The Richards model was defined as [$\mathrm{OD}\left(t\right)=y_{0}+\frac{A}{{\left[1+\exp\left\{-r\left(t-t_{\mathrm{mid}}\right)\right\}\right]}^{1/\nu }}$], where νis a shape parameter controlling curve asymmetry. The Baranyi–Roberts model was defined as [$\mathrm{OD}\left(t\right)=y_{0}+A\times \frac{\log\left\{1+\exp\left[\mu \left(t-\lambda \right)\right]-\exp\left(-\mu \lambda \right)\right\}}{\log\left\{1+\exp\left[\mu \left(t_{\max}-\lambda \right)\right]-\exp\left(-\mu \lambda \right)\right\}}$], where $t_{\max}$is the maximum observed time point.

Model fitting was performed separately for each strain curve using nonlinear least squares. Model performance was compared using Akaike information criterion (AIC), [$\mathrm{AIC}=2k-2\ln\left(L\right)]$, Bayesian information criterion (BIC) [$\mathrm{BIC}=k\ln\left(n\right)-2\ln\left(L\right)]$, and root mean squared error (RMSE), [$\mathrm{RMSE}=\sqrt{\frac{1}{n}\sum_{i=1}^{n}{\left(OD_{\mathrm{obs},i}-OD_{\mathrm{pred},i}\right)}^{2}}$], where kis the number of estimated parameters, nis the number of observations, Lis the model likelihood, $OD_{\mathrm{obs},i}$is the observed OD, and $OD_{\mathrm{pred},i}$is the model-predicted OD. The best-fitting model for each strain specific growth curve was selected based on the lowest BIC. Strain-specific parameter estimates were retained for visualization and then summarized within strain for comparisons with MIC values, serotype, and AMR genes.

The primary parameters interpreted were $y_{0}$, A, μ, and λ. Here, $y_{0}$represents the initial or baseline OD, Arepresents stationary maximum growth yield, μrepresents maximum growth rate, and λrepresents estimated lag time. These parameters were interpreted as descriptors of growth fitness characteristics under the tested *in vitro* conditions**.**

### 2.7 Statistical analysis

Growth fitness parameters, including intrinsic growth rate, maximum stationary-phase density, and lag duration, were compared across ceftriaxone and ciprofloxacin MIC categories, AMR genotypes, serovars, and MDR status (resistant to ≥3 antimicrobial classes). Associations between antimicrobial susceptibility, AMR genotype, and bacterial growth parameters were evaluated using linear mixed-effects models. MIC values were analyzed both as categorical variables and as continuous log₂-transformed predictors, such that each one-unit increase represented a two-fold increase in MIC. For each growth parameter, including intrinsic growth rate, lag-phase duration, and maximum stationary-phase density, models were fitted using *lmer*(), with strain included as a random intercept to account for technical triplicates. Fixed effects included MIC category or log₂-transformed MIC and retail meat source. The significance of fixed effects was assessed using Type III analysis of variance with Satterthwaite's approximation. When the overall MIC effect was significant, pairwise comparisons among MIC categories were performed using estimated marginal means with Tukey adjustment for multiple comparisons. Spearman rank correlations were also used to assess associations between MIC values, or the total number of AMR genes per strain, and strain-averaged growth parameters, with *P*-values adjusted for multiple testing using the Benjamini-Hochberg method [20].

Associations between AMR genotype and growth parameters were evaluated by grouping strains according to β-lactam resistance gene profiles for ceftriaxone-related comparisons and quinolone or fluoroquinolone resistance mechanisms for ciprofloxacin-related comparisons. Strains without detected resistance determinants in the relevant antimicrobial class were classified as “None,” whereas low-frequency genotype groups, defined as those present in ≤2 strains, were collapsed into an “other” category. Differences in growth parameters among genotype groups were tested using linear mixed-effects models with genotype group and retail meat source as fixed effects and strain as a random intercept. The same modeling framework was used to assess associations between growth parameters and MDR status and serovar group, with serovars represented by ≤2 strains collapsed into an “other” category. Estimated marginal means and 95% confidence intervals were reported for all models. Bootstrap resampling with 5000 iterations was used to summarize median intrinsic growth rates and 95% percentile intervals for each genotype group.

All statistical analyses were performed in R (version 4.3; R Core Team, 2023) using the packages lme4 [21], lmerTest [22], and emmeans [23].

## 3 Results

### 3.1 Antimicrobial resistance was not associated with measurable reductions in intrinsic growth rate in retail meat–associated *Salmonella enterica*

We evaluated whether ceftriaxone and ciprofloxacin susceptibility profiles were associated with differences in in vitro growth fitness using a panel of 25 nontyphoidal *S. enterica* isolates. These isolates were collected from U.S. retail chicken, turkey, pork, and beef and represented diverse serovars (Table 1 and Table S1). The collection spanned a wide range of MICs, with ceftriaxone MICs ranging from ≤0.25 to 64 μg/mL and ciprofloxacin MICs from ≤0.015 to 1 μg/mL. Growth curves of all strains followed the expected sigmoidal shape (Fig. 1 A and B; and Fig. S1). As expected, negative control wells (media only) showed no detectable growth, whereas the internal control strain exhibited consistent growth kinetics (Fig. S1). Among several mathematical models tested, the Gompertz model provided the best overall fit (Fig. S2). Therefore, growth fitness parameters—including intrinsic growth rate, maximum density and lag duration were derived from the Gompertz model and compared according to MIC values, resistance genotypes, and serovars.

**Fig. 1:**
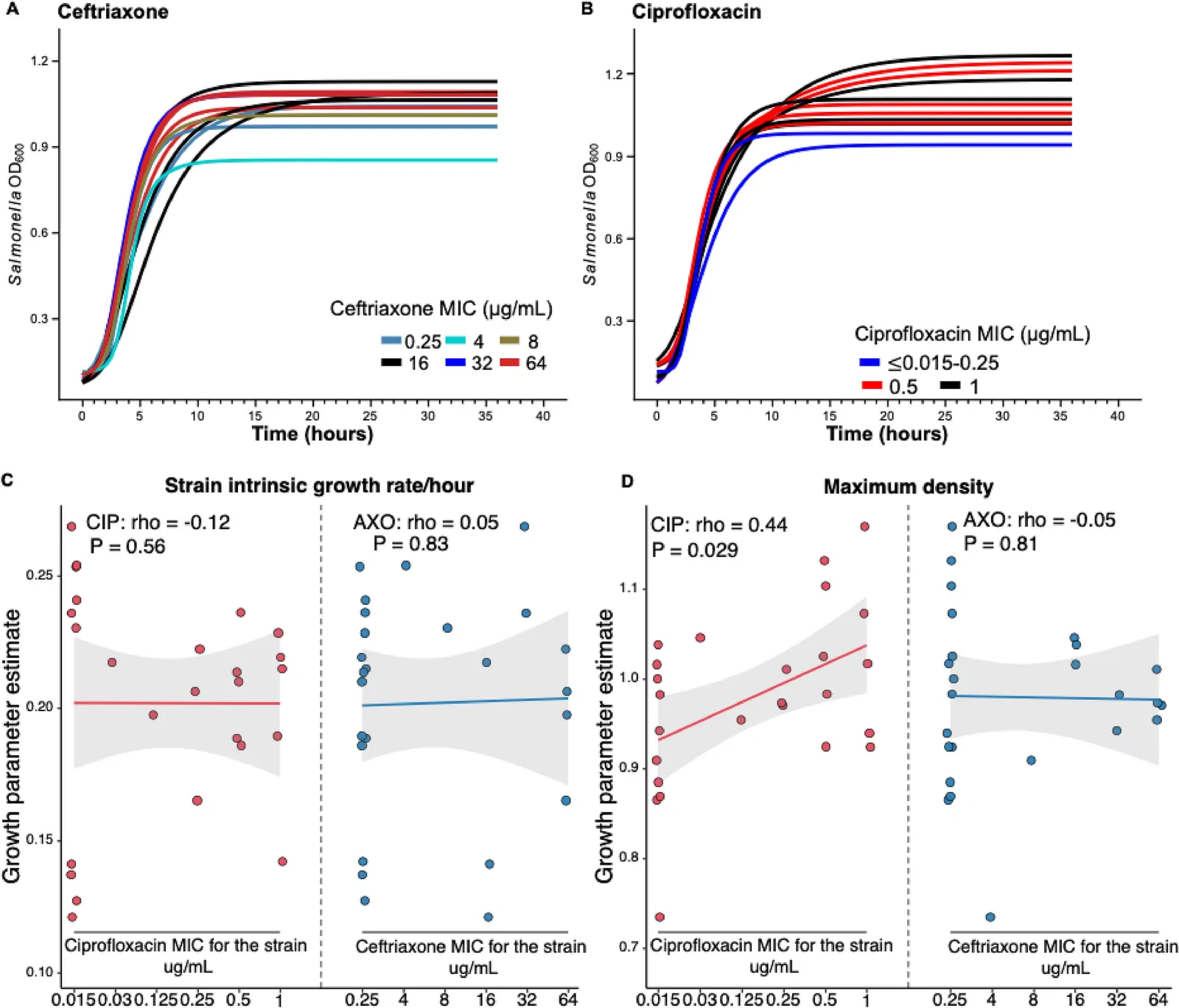
Growth fitness of *S. enterica* strains across ceftriaxone and ciprofloxacin minimum inhibitory concentrations (MICs). (A–B) Model-predicted growth curves of strains with varying ceftriaxone (A) and ciprofloxacin (B) MICs. Each line represents the strain-level mean predicted growth trajectory derived from the best-fitting nonlinear growth model, with colors indicating MIC categories. (C—D) Relationships between ciprofloxacin (CIP) and ceftriaxone (AXO) MICs and intrinsic growth rate (C) or maximum stationary-phase density (D). Points represent strain-level estimates, and solid lines indicate fitted trends with shaded 95% confidence intervals. Spearman's correlation coefficients (ρ) and corresponding adjusted *P* values are displayed in each panel.

Despite the range of MIC values, predicted growth trajectories were broadly similar across strains, with similar growth fitness distributions across the standard two-fold MIC dilutions for either ceftriaxone- or ciprofloxacin-resistant strains (Fig. 1C). For example, Spearman correlation analyses showed no relationship between intrinsic growth rate and MIC for either drug (ciprofloxacin ρ = −0.12, *P* = 0.56; ceftriaxone ρ = −0.05, *P* = 0.56), nor with initial density (both *P* > 0.05). However, in the ciprofloxacin dataset, we observed a positive correlation with stationary-phase maximum density (ρ = 0.43, *P* = 0.035), which meant that as MIC increased, so did maximum density (Fig. 1D, Fig. S3).

Generalized linear mixed model analyses further supported these trends when MIC was modeled as a categorical factor. Although strains with susceptible or reduced-susceptibility profiles to ceftriaxone exhibited slightly higher average intrinsic growth rates than those in the ciprofloxacin dataset (0.220/h, 95% CI, 0.184–0.255 vs. 0.200/h, 95% CI, 0.160–0.246), MIC category was not significantly associated with intrinsic growth rate for either drug (LMM ceftriaxone: *P* = 0.166; ciprofloxacin: *P* = 0.996). Maximum density and lag duration likewise showed no relationship with MIC category. When MIC was modeled as a log₂-transformed continuous predictor, we again did not observe associations between MIC and growth rate for either drug (ciprofloxacin β = −0.0013, *P* = 0.0761; ceftriaxone β = −0.0004, *P* = 0.877), nor with other growth fitness parameters (Table S2). When strains were grouped by susceptibility category (i.e., susceptible [S] and resistant [R]), *S. enterica* showed comparable intrinsic growth rates for both ciprofloxacin and ceftriaxone (Fig. S4). Initial MIC values were determined by the standard broth microdilution method, which reports antimicrobial susceptibility in two-fold dilution increments. Because this approach can yield relatively wide MIC intervals, we retested a subset of strains using *E*-tests®, which provide a more continuous estimate of MIC for ceftriaxone and ciprofloxacin. However, E-test MICs were also not correlated with strain-averaged intrinsic growth rate for either drug (*P* > 0.05). Likewise, viable bacterial counts showed comparable starting CFU/mL and similar stationary-phase counts across MIC categories (Fig. S5). These findings suggest that resistance to these clinically important antimicrobials is not consistently associated with reduced growth rates and, in some cases, may be associated with increased maximum density or higher population yield. Together, the results indicate that varying levels of phenotypic susceptibility to either ceftriaxone or ciprofloxacin among retail meat–associated *S. enterica* strains were not consistently associated with reductions in intrinsic growth rate.

### 3.2 Presence of β-lactam and fluoroquinolone resistance mechanisms is not associated with reduced intrinsic growth fitness in *Salmonella enterica*

We next evaluated whether contemporary AMR genotypes led to differences in growth fitness in addition to phenotypic susceptibility as measured by MIC (Fig. 2). The strain set included isolates with and without mechanisms associated with reduced susceptibility or resistance to β-lactam/penicillin and fluoroquinolone antimicrobials, including *bla*_CMY-2_, *bla*_CTX-M-65_, *bla*_TEM-1_, *qnrB19*, *gyrA* D87Y mutation, and other low-prevalence β-lactam/penicillin resistance genes detected in only one or two strains (Table 1 and Table S1). Although strains without resistance mechanisms exhibited numerically higher average intrinsic growth rates than those encoding resistance determinants, we did not find enough evidence for statistical differences between strains with and without *qnr* or *gyrA* genotypes (*P* = 0.970), nor between strains with and without *bla* genotypes (*bla*_CMY-2_, *bla*_TEM-1_, both *bla*_CMY-2_ and *bla*_TEM-1_, or both *bla*_CTX-M-65_ and *bla*_TEM-1_; *P* = 0.9871) (Fig. 2A and B). Genotype was also not associated with maximum stationary-phase density or lag duration for either ciprofloxacin or ceftriaxone (maximum stationary-phase density: *P* = 0.444 and *P* = 0.723; lag duration: *P* = 0.728 and *P* = 0.634).

**Fig. 2:**
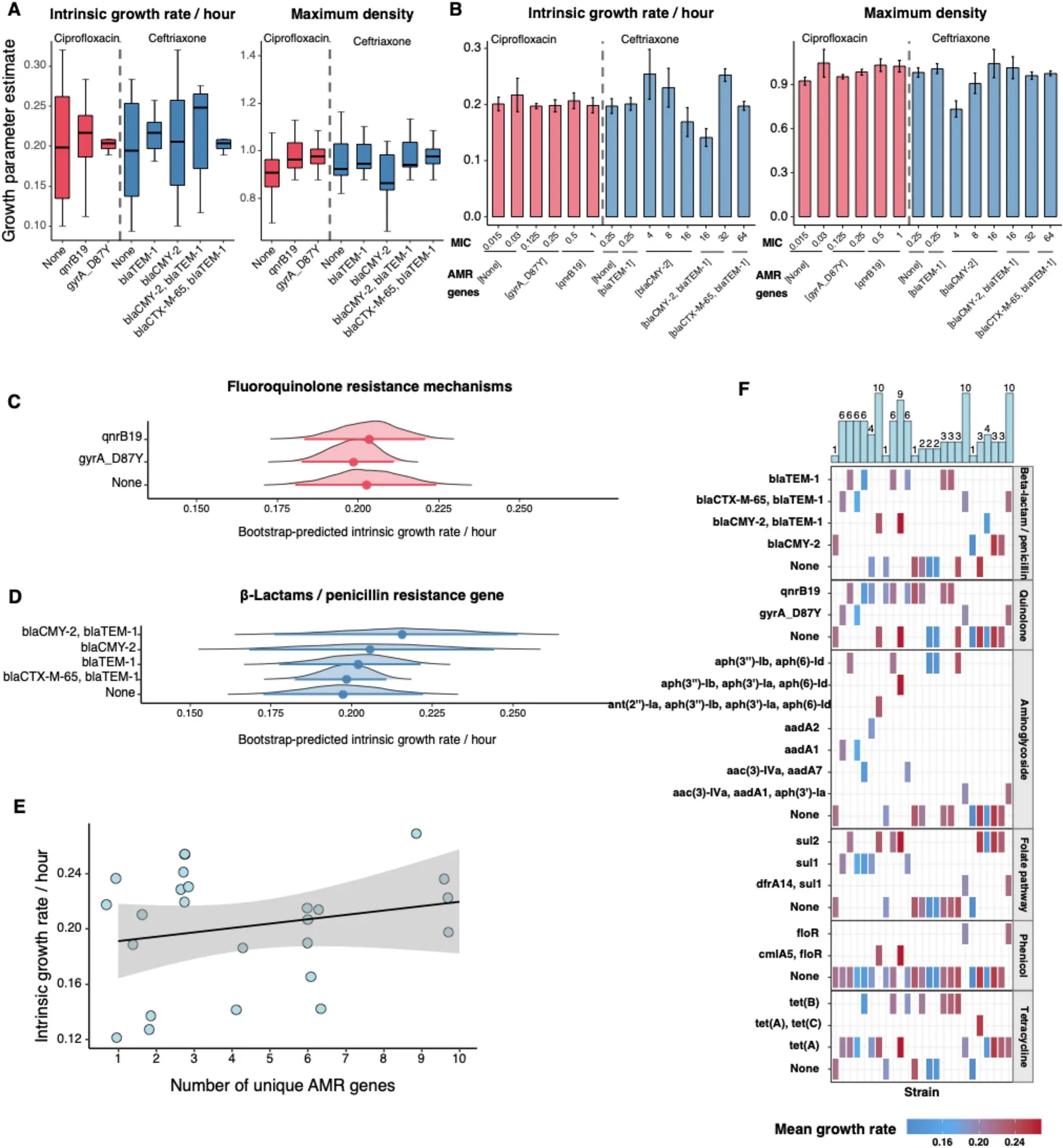
Growth fitness characteristics of *S. enterica* strains with different ceftriaxone- and ciprofloxacin-associated resistance genotypes. (A–B) Boxplots showing the distributions of intrinsic growth rate and maximum stationary-phase density (A), and intrinsic growth rate and maximum density across minimum inhibitory concentration (MIC) categories (B), among strains with different β-lactam and fluoroquinolone resistance mechanisms. “None” indicates strains without detected resistance determinants associated with the corresponding antimicrobial class. (C—D) Bootstrap-predicted distributions of intrinsic growth rates among strains grouped by fluoroquinolone resistance mechanisms (C) and β-lactam/penicillin resistance genes (D). Ridgelines represent bootstrap distributions, points indicate median estimates, and horizontal lines represent 95% bootstrap intervals. (E) Association between the number of unique antimicrobial resistance (AMR) genes and intrinsic growth rate. The solid line represents the fitted linear trend, with the shaded area indicating the 95% confidence interval. (F) Heatmap showing AMR gene profiles across *S. enterica* strains ordered by intrinsic growth rate. Columns represent individual strains, and rows represent AMR genes grouped by antimicrobial class. Tile colors indicate strain-specific intrinsic growth rate for detected genes, whereas blank cells indicate genes not detected in the corresponding strain. The bar plot above the heatmap shows the number of unique AMR genes per strain.

To further assess genotype-level variability in growth fitness, we performed bootstrap resampling with 5000 iterations of intrinsic growth-rate distributions for these genotypes. The resulting distributions were highly similar across genotypes (Figs. 2C and D). For example, projected median growth rates ranged from 0.199 to 0.203/h for strains carrying *qnrB19* or *gyrA* D87Y, and from 0.197 to 0.216/h for strains carrying *bla*_CMY-2_, *bla*_TEM-1_, or *bla*_CTX-M-65_ genes.

The strains also encoded genes associated with resistance to other antimicrobial classes, including aminoglycosides, phenicols, and tetracyclines (Table 1 and Table S1). This includes aminoglycoside genes (*aac*(3)-IVa, *aadA1*, *aadA7*, *aph*(3″)-Ib, *aph*(6)-Id), folate-pathway genes (*sul1*, *sul2*, *dfrA14*), phenicol genes (*floR*, *cmlA5*), and tetracycline genes (*tet*(A), *tet*(B), *tet*(C)). On average, each resistant strain carried four unique AMR genes (range: 1–10) (Figs. 2E and F). Although higher AMR gene count showed a modest positive association with growth rate, we found no significant linear relationship between AMR gene richness and intrinsic growth rate or any other growth fitness parameter (*P* = 0.373) (Fig. 2F, Table 1 and Table S3). These findings do not demonstrate a consistent association between the number of AMR genes (across different drug classes) and differences in growth fitness traits in these *S. enterica* strains (Fig. S6). Together, these findings show that the presence of AMR genes, including multi-class AMR gene profiles, did not lead to detectable variation in the growth fitness of *S. enterica* from retail meat under the tested in vitro conditions.

### 3.3 Multidrug-resistant *Salmonella enterica* serovars retain comparable in vitro growth fitness despite presence of clinically important resistance genes

We next assessed whether MDR status or the presence of clinically relevant resistance genes was associated with differences in in vitro growth fitness among *S. enterica* serovars (Fig. 3). The data showed that strains resistant to ≤1–3 antimicrobial classes had a slightly higher mean intrinsic growth rate than MDR strains, although this difference was not statistically significant (0.214/h, 95% CI, 0.180–0.248 vs. 0.207/h, 95% CI, 0.177–0.237, Fig. 3A and B). Maximum stationary-phase density was also comparable between these groups (0.976, 95% CI, 0.895–1.03 vs. 0.982, 95% CI, 0.921–1.04, Fig. 3C, Table S4). These data indicate that cumulative phenotypic resistance across antimicrobial classes was not associated with a measurable reduction in growth fitness under the conditions tested.

**Fig. 3:**
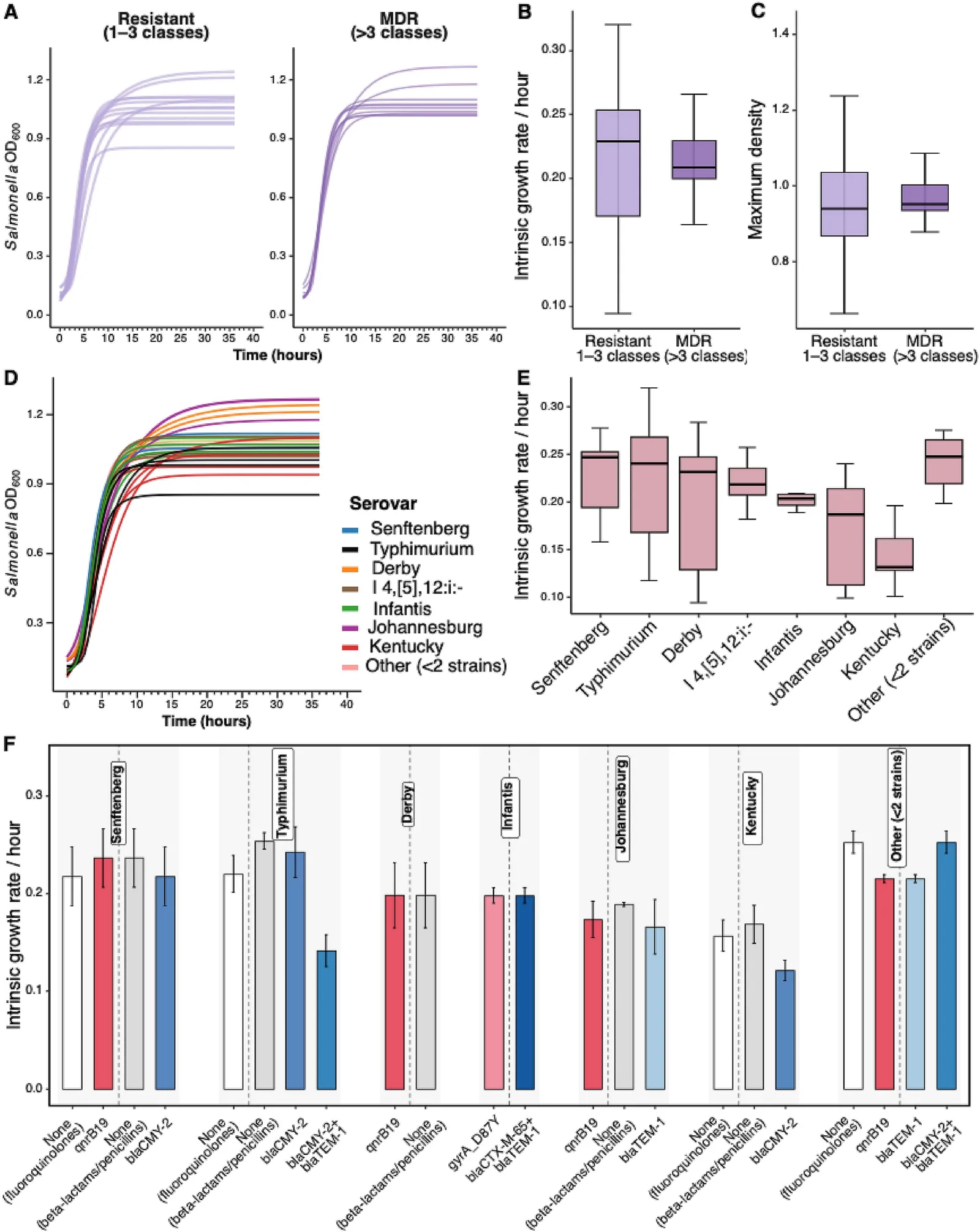
Growth fitness of *S. enterica* strains across multidrug resistance status, serovars, and antimicrobial resistance genotypes. (A) Model-predicted growth curves of strains grouped by multidrug resistance (MDR) status (resistance to ≥3 antimicrobial classes). Each line represents the strain-level mean predicted growth trajectory. (B—C) Boxplots showing the distributions of intrinsic growth rate (B) and maximum stationary-phase density (C) among non-MDR (resistance to <3 antimicrobial classes) and MDR strains. (D) Model-predicted growth curves of strains grouped by serovar, with each line representing the strain-level mean predicted growth trajectory. (E) Boxplots showing the distribution of intrinsic growth rates across *S. enterica* serovars. Serovars represented by fewer than two strains were grouped into the “Other” category. (F) Intrinsic growth rates of strains grouped by serovar and antimicrobial resistance genotype. Bars represent strain-level growth rate estimates, with colors indicating the corresponding resistance genotype categories. “None” indicates no detected resistance determinants associated with the antimicrobial class being evaluated.

Growth fitness varied modestly across serovars with different AMR phenotypes and resistance gene profiles, with one to four isolates of each of the serotypes (Fig. 3D and E). For example, serotype Derby had the highest intrinsic growth rate, followed by I 4, [5],12:i:-, whereas Kentucky had the lowest growth rate. Maximum stationary-phase density was numerically highest for Derby and Johannesburg, while lag duration was longest for Typhimurium and shortest for Kentucky. However, no serovar showed consistently reduced growth fitness across all measured parameters, suggesting that susceptibility phenotype or the presence of resistance genes did not necessarily lead to variation in the evaluated growth fitness traits.

When we further examined AMR genotype profiles within individual serovars, we observed that intrinsic growth rates were also similar across isolates harboring fluoroquinolone and β-lactam resistance mechanisms, with only small numerical differences (Fig. 3F). For example, *S. enterica* serotype Senftenberg isolates with and without the fluoroquinolone resistance gene *qnrB19* had nearly identical mean growth rates (0.225 vs. 0.226/h). Similarly, Typhimurium serotype harboring *bla*_CMY-2_, *bla*_TEM-1_, or both genes showed only small differences in growth rate relative to isolates without these β-lactamase genotypes.

Notably, the fluoroquinolone resistance mutation *gyrA* D87Y was detected only in the Infantis serotype, where it co-occurred with the extended-spectrum cephalosporin resistance gene *bla*_CTX-M-65_. These Infantis isolates, recovered from chicken or turkey meat, were phenotypically resistant to both ciprofloxacin and ceftriaxone yet retained an intrinsic growth rate of 0.198/h (Fig. 3F, Fig. S7). Similarly, the monophasic Typhimurium variant *S. enterica* serovar I 4, [5],12:i:- harboring *qnrB19* showed growth estimates comparable to other serovars. Interestingly, phenicol resistance genes (*catA1* and *catB3*) were also detected in this serotype. Kentucky serovar isolates harboring *bla*_CMY-2_ had only a slightly lower estimated growth rate than isolates of the same serotype without detected fluoroquinolone or β-lactam/penicillin resistance genes (0.155 vs. 0.159/h) (Fig. 3F).

## 4 Discussion

In this study, contemporary nontyphoidal *S. enterica* strains with reduced susceptibility or clinical resistance to ceftriaxone or ciprofloxacin did not consistently show reduced intrinsic growth rate or other growth fitness traits. This pattern was also observed across AMR genotypes, MDR status, and serovars. In some cases, reduced ciprofloxacin susceptibility was associated with modestly increased maximum population density. These findings suggest that phenotypic AMR and the cumulative burden of AMR genes in foodborne *S. enterica* are not necessarily accompanied by a measurable growth disadvantage.

Strains carrying β-lactam resistance genes, including *bla*_CMY-2_ and *bla*_CTX-M-65_, or fluoroquinolone resistance mechanisms, including *qnrB19* and the *gyrA* D87Y mutation, generally had growth rates comparable to strains lacking these determinants. Of particular concern were serovar *Infantis* isolates from chicken and turkey meat that carried both *bla*_CTX-M-65_ and the *gyrA* D87Y mutation. Recent whole-genome sequencing studies have also highlighted MDR *S. enterica* serovar *Infantis* as an emerging public health concern [24]. In our study, these isolates were phenotypically resistant to both ceftriaxone and ciprofloxacin but retained growth fitness comparable to serovars without these resistance determinants. Because *bla*_CTX-M_ genes are often plasmid-associated and may co-occur with resistance determinants for multiple antimicrobial classes, these strains may have the capacity to persist and disseminate without an apparent growth disadvantage [25]. However, the absence of a detectable growth fitness cost does not imply that AMR has no biological cost. Rather, it highlights the complexity of AMR associated fitness effects [25], which may depend on strain background, resistance mechanism, plasmid adaptation, co-selection, and compensatory evolution [11], [25]. Thus, reducing antimicrobial use may limit further selection for resistance, but it may not rapidly eliminate well-adapted resistant strains, particularly when resistance determinants occur in genomic contexts or plasmids that minimize associated bacterial fitness costs.

These results have important implications for AMR surveillance and stewardship. In retail meat surveillance, identifying resistant *Salmonella* highlights strains of clinical concern, whereas evaluating growth fitness provides additional insight into whether those strains may persist and compete with susceptible strains. Importantly, while antimicrobial use provides strong selective pressure for resistance determinants, our in vitro results suggest that some resistant isolates can maintain growth characteristics similar to susceptible isolates in the absence of antimicrobial selection. These findings do not establish how antimicrobial stewardship will affect AMR prevalence, but suggest that the persistence of well-adapted resistant populations may complicate reductions in resistance. This has been observed in some prior instances, such as in response to the FDA removing approval for fluoroquinolones in poultry, which stemmed the increase in resistant *Campylobacter* but did not result in an immediate or large decrease in resistance prevalence [26].

This study should be interpreted in the context of its experimental design. The growth fitness in this study was assessed in monoculture under aerobic laboratory conditions and may not fully reflect fitness under microbial competition [27]. Therefore, these findings should be interpreted as evidence of comparable in vitro growth fitness, rather than direct evidence of persistence in vivo or in the food chain. In addition, the relatively low number of strains and few strains per serotype limit wide-ranging conclusions as to their applicability to all *Salmonella*. Nevertheless, the study included strains from multiple retail meat sources representing diverse serovars, phenotypic and genotypic antimicrobial resistance profiles, and MDR status. Growth fitness was evaluated using mathematical growth-curve modeling to estimate multiple quantitative parameters, including maximum growth rate, lag time, and growth yield, while complementary statistical analyses examined their associations with phenotypic and genotypic resistance characteristics. Furthermore, phenotypic antimicrobial susceptibility was initially assessed using standard broth microdilution, followed by gradient diffusion *E*-tests for selected strains to obtain more refined MIC estimates for ceftriaxone and ciprofloxacin. Together, these approaches provided a more comprehensive assessment of the magnitude and consistency of growth fitness differences rather than relying solely on statistical significance. However, these analytical strengths do not overcome the limitations of the small sample size, particularly the reduced statistical power to detect modest effects within individual serovars or resistance categories, or reflect potential heterogeneity within serotypes. These findings highlight the importance of continued AMR surveillance integrating phenotypic and genomic data to monitor resistant *Salmonella* populations and understand the potential broader significance of our findings. Future studies using larger surveillance collections and ecologically relevant conditions will be important to determine whether resistant *S. enterica* strains that maintain growth fitness in vitro are also more likely to persist or disseminate across the food chain.

## 5 Conclusion

In this study, we combined genomic and phenotypic analyses to evaluate the in vitro growth fitness of retail meat associated *S. enterica* isolates with reduced susceptibility or resistance to ceftriaxone and ciprofloxacin. Resistant *S. enterica* strains did not consistently exhibit reduced intrinsic growth rates and generally maintained growth fitness comparable to that of susceptible strains. From a One Health perspective, these findings suggest that resistance was not consistently associated with a detectable growth disadvantage under the tested in vitro conditions. This underscores the need for strengthened integrated surveillance, genomic monitoring, and prudent antimicrobial use in food-animal production to limit the spread of resistance.

## CRediT authorship contribution statement

**Maria Camila Duarte-Villanueva:** Writing – original draft, Methodology, Investigation, Data curation. **Gregory H. Tyson:** Writing – review & editing, Resources, Methodology, Investigation. **Tara N. Gaire:** Writing – review & editing, Writing – original draft, Supervision, Resources, Project administration, Methodology, Investigation, Funding acquisition, Formal analysis, Conceptualization.

## Ethics statement

No ethical approval was required for this research work.

## Disclaimer

The views expressed in this article are those of the authors and do not necessarily reflect the official policy of the Department of Health and Human Services, the U.S. Food and Drug Administration, or the U.S. Government.

## Funding

This work was supported by the 10.13039/100007131Texas Tech University School of Veterinary Medicine, Amarillo, Texas, 79106, USA.

## Declaration of competing interest

The authors declare no competing interests.

## Data Availability

The datasets generated and analyzed during the current study are available from the supplementary file and further corresponding author upon reasonable request.
