# Supplementary material for: Impact of antimicrobial resistance genes and phenotypic susceptibility profiles on growth fitness in nontyphoidal Salmonella enterica from U.S. retail meat surveillance
Source: One Health. 2026 Oct 30;23:101642. doi: 10.1016/j.onehlt.2026.101642 (PMC13770629; doi:10.1016/j.onehlt.2026.101642)
Supplement: Supplementary file 1 [file mmc1.docx]

**Supplemental information**

**Supplemental Table**

**Table S1.** **Table S1. Characteristics of *Salmonella enterica* strains isolated from retail meat through the FDA National Antimicrobial Resistance Monitoring System.** The table summarizes strain identifiers, serovars, isolation years, retail meat sources, host species, ceftriaxone and ciprofloxacin MICs and susceptibility classifications, antimicrobial non-susceptibility profiles, detected antimicrobial resistance, and BioSample accession numbers.

**Table S2. Association between antimicrobial MICs and growth fitness parameters.** Linear regression estimates describing the associations between log₂-transformed MIC values and model-derived growth parameters for ceftriaxone and ciprofloxacin. Slope estimates (β) represent the expected change in each growth parameter associated with a two-fold increase in MIC. Results are presented as estimates with 95% confidence intervals (CIs) and P values.

**Table S3. Association between AMR gene burden and growth fitness parameters in *Salmonella enterica*.** Linear regression estimates describing the relationships between the number of unique AMR genes detected per strain and model-derived intrinsic growth rate, maximum density, and lag duration. Results are presented with 95% confidence intervals (CIs) and P values.

**Table S4. Differences in growth fitness parameters by antimicrobial resistance-class group.** Estimated differences in model-derived growth parameters between non-multidrug-resistant (non-MDR) and multidrug-resistant (MDR) Salmonella enterica strains. MDR was defined as resistance to three or more antimicrobial classes. Estimates represent differences between resistance groups, with 95% confidence intervals (CIs) and P values reported for each growth parameter.

**Supplemental Figure**

**Figure S1. Strain-specific in vitro growth dynamics of *Salmonella enterica*.** Corrected OD600 values were plotted over time for each isolate and control. Solid black lines show LOESS-smoothed growth trajectories, and dotted lines represent 95% confidence intervals. Most S. enterica isolates displayed sigmoidal growth patterns, with isolate-level variation in lag phase, growth rate, and maximum OD600. The negative control remained near baseline, whereas the internal control strain (Escherichia coli ATCC 1430) exhibited consistent growth kinetics.

**Figure S2. BIC model comparison for bacterial growth curves across *Salmonella enterica* strains and replicates.** For each strain–replicate combination, Bayesian information criterion (BIC) values are shown for five candidate growth models (constant, logistic, Gompertz, Richards, and Baranyi–Roberts). Lower BIC values indicate better model fit, accounting for model complexity. Bars indicate the best-fitting model for each strain–replicate combination (i.e., the model with the lowest BIC). The bottom row shows results for the negative control (media only) and the internal control strain (Escherichia coli ATCC 1430).

**Figure S3. Spearman correlations between antimicrobial susceptibility and growth fitness parameters in *Salmonella enterica*.** Scatter plots show associations between MIC values and model-derived maximum density, intrinsic growth rate, and initial density for ceftriaxone and ciprofloxacin. Points represent individual strains, and solid lines show fitted trends for visualization. Spearman correlation coefficients and adjusted P values are shown within each panel. Red points indicate ciprofloxacin, and blue points indicate ceftriaxone.

**Figure S4. Growth fitness of retail meat–associated *Salmonella enterica* strains by antimicrobial susceptibility category.** Boxplots show the distributions of intrinsic growth rate, lag duration, and maximum density for isolates categorized as susceptible or resistant/reduced susceptibility to ceftriaxone and ciprofloxacin. Susceptibility categories were defined using MIC breakpoints, with ceftriaxone resistance defined as MIC ≥ 4 µg/mL and ciprofloxacin susceptibility defined as MIC ≤ 0.05 µg/mL.

**Figure S5. Bacterial counts of *Salmonella enterica* strains across antimicrobial susceptibility, genotype, and serovar groups.** Bars show mean bacterial counts expressed as log₁₀ CFU/mL, and error bars indicate standard errors. Panels show 24-h bacterial counts across ciprofloxacin and ceftriaxone MICs; bacterial counts at 0 and 24 h across MICs and serovars; 24-h bacterial counts by multidrug-resistance status and resistance genotype; and 24-h bacterial counts by serovar within fluoroquinolone and β-lactam resistance gene groups.

**Figure S6. Relationship between AMR gene burden and growth fitness parameters in *Salmonella enterica*.** Scatter plots show the relationships between the number of unique AMR genes per strain and model-derived intrinsic growth rate, maximum density, and lag duration. Points represent individual strains, solid lines show fitted linear trends for visualization, and shaded areas indicate 95% confidence intervals.

**Figure S1. Strain-specific in vitro growth dynamics of *Salmonella enterica*.** Corrected OD600 values were plotted over time for each isolate and control. Solid black lines show LOESS-smoothed growth trajectories, and dotted lines represent 95% confidence intervals. Most S. enterica isolates displayed sigmoidal growth patterns, with isolate-level variation in lag phase, growth rate, and maximum OD600. The negative control remained near baseline, whereas the internal control strain (Escherichia coli ATCC 1430) exhibited consistent growth kinetics.

 **Figure S2. BIC model comparison for bacterial growth curves across *Salmonella enterica* strains and replicates.** For each strain–replicate combination, Bayesian information criterion (BIC) values are shown for five candidate growth models (constant, logistic, Gompertz, Richards, and Baranyi–Roberts). Lower BIC values indicate better model fit, accounting for model complexity. Bars indicate the best-fitting model for each strain–replicate combination (i.e., the model with the lowest BIC). The bottom row shows results for the negative control (media only) and the internal control strain (Escherichia coli ATCC 1430).

**Figure S3. Spearman correlations between antimicrobial susceptibility and growth fitness parameters in *Salmonella enterica*.** Scatter plots show associations between MIC values and model-derived maximum density, intrinsic growth rate, and initial density for ceftriaxone and ciprofloxacin. Points represent individual strains, and solid lines show fitted trends for visualization. Spearman correlation coefficients and adjusted P values are shown within each panel. Red points indicate ciprofloxacin, and blue points indicate ceftriaxone.

**Figure S4. Growth fitness of retail meat–associated *Salmonella enterica* strains by antimicrobial susceptibility category.** Boxplots show the distributions of intrinsic growth rate, lag duration, and maximum density for isolates categorized as susceptible or resistant/reduced susceptibility to ceftriaxone and ciprofloxacin. Susceptibility categories were defined using MIC breakpoints, with ceftriaxone resistance defined as MIC ≥ 4 µg/mL and ciprofloxacin susceptibility defined as MIC ≤ 0.05 µg/mL.

**Figure S5. Bacterial counts of *Salmonella enterica* strains across antimicrobial susceptibility, genotype, and serovar groups.** Bars show mean bacterial counts expressed as log₁₀ CFU/mL, and error bars indicate standard errors. Panels show 24-h bacterial counts across ciprofloxacin and ceftriaxone MICs; bacterial counts at 0 and 24 h across MICs and serovars; 24-h bacterial counts by multidrug-resistance status and resistance genotype; and 24-h bacterial counts by serovar within fluoroquinolone and β-lactam resistance gene groups.

**Figure S6. Relationship between AMR gene burden and growth fitness parameters in *Salmonella enterica*.** Scatter plots show the relationships between the number of unique AMR genes per strain and model-derived intrinsic growth rate, maximum density, and lag duration. Points represent individual strains, solid lines show fitted linear trends for visualization, and shaded areas indicate 95% confidence intervals.

**Supplementary Table**

**Table S1. Characteristics of *Salmonella enterica* strains isolated from retail meat through the FDA National Antimicrobial Resistance Monitoring System.** The table summarizes strain identifiers, serovars, isolation years, retail meat sources, host species, ceftriaxone and ciprofloxacin MICs and susceptibility classifications, antimicrobial non-susceptibility profiles, detected antimicrobial resistance (AMR), and BioSample accession numbers.

| **No.** | **NARMS ID** | **Serovar** | **Year** | **Retail source** | **Host** | **Ceftriaxone MIC* (SIR)** | **Ciprofloxacin MIC (SIR)** | **Non-susceptible profile** | **AMR determinants** | **BioSample ID** |
| --- | --- | --- | --- | --- | --- | --- | --- | --- | --- | --- |
| 1 | N21S0024 | Kentucky | 2021 | Chicken | Chicken | ≤0.25 (S) | ≤0.015 (S) | None detected | aph(3'')-Ib, aph(6)-Id | SAMN38156043 |
| 2 | N21S0164 | I 4,[5],12:i:- | 2021 | Chicken | Chicken | ≤0.25 (S) | 1 (R) | AMP R, CIP R, NAL R, TET R | *bla*TEM-1, *qnrB19*, *tet*(B) | SAMN38156109 |
| 3 | N21S0166 | I 4,[5],12:i:- | 2021 | Ground turkey | Turkey | ≤0.25 (S) | 1 (R) | AMP R, CIP R, NAL R, TET R | *bla*TEM-1, *qnrB19*, *tet*(B) | SAMN18685282 |
| 4 | N21S0184 | Kentucky | 2021 | Chicken | Chicken | ≤0.25 (S) | ≤0.015 (S) | TET R | aph(3'')-Ib, aph(6)-Id, *tet*(B) | SAMN19314170 |
| 5 | N21S0269 | Typhimurium | 2021 | Chicken | Chicken | 4 (R) | ≤0.015 (S) | AMC R, AMP R, AXO R, FIS R, FOX R, SUF R, TET R | *bla*CMY-2, *sul2*, *tet*(A) | SAMN19115655 |
| 6 | N21S0295 | Typhimurium | 2021 | Chicken | Chicken | 8 (R) | ≤0.015 (S) | AMC R, AMP R, AXO R, FIS R, FOX R, SUF R, TET R | *bla*CMY-2, *sul2*, *tet*(A) | SAMN40541415 |
| 7 | N21S0025 | Kentucky | 2021 | Chicken | Chicken | ≤0.25 (S) | ≤0.015 (S) | None detected | aph(3'')-Ib, aph(6)-Id | SAMN38156044 |
| 8 | N21S0266 | Typhimurium | 2021 | Chicken | Chicken | 16 (R) | ≤0.015 (S) | AMC R, AMP R, AXO R, FIS R, FOX R, SUF R, TET R | *bla*CMY-2, *sul2*, *bla*TEM-1, *tet*(A) | SAMN19115653 |
| 9 | N21S0244 | Kentucky | 2021 | Chicken | Chicken | 16 (R) | ≤0.015 (S) | AMC R, AMP R, AXO R, FOX R | *bla*CMY-2 | SAMN19605052 |
| 10 | N21S0245 | Typhimurium | 2021 | Chicken | Chicken | ≤0.25 (S) | ≤0.015 (S) | FIS R, SUF R, TET R | *sul2*, *tet*(A), *tet*(C) | SAMN19605053 |
| 11 | N20S0277 | Derby | 2020 | Pork chops | Swine | ≤0.25 (S) | 0.5 (I) | CIP I, NAL R, TET R | *fosA7.3*, *qnrB19*, *tet*(B) | SAMN19221120 |
| 12 | N19S1442 | Johannesburg | 2019 | Pork chops | Swine | ≤0.25 (S) | 0.5 (I) | CIP I | *qnrB19* | SAMN15377807 |
| 13 | N19S0182 | Senftenberg | 2019 | Ground turkey | Turkey | 16 (R) | 0.03 (S) | AMC R, AMP R, AXO R, FOX R | *bla*CMY-2 | SAMN12842790 |
| 14 | N19S1464 | I 4,[5],12:i:- | 2019 | Ground beef | Cattle | ≤0.25 (S) | 0.5 (I) | AMP R, CIP I, FIS R, NAL R, SUF R, TET R | aph(3'')-Ib, aph(6)-Id, *sul2*, *bla*TEM-1, *qnrB19*, *tet*(B) | SAMN13266253 |
| 15 | N19S0868 | Derby | 2019 | Pork chops | Swine | ≤0.25 (S) | 0.5 (I) | CIP I, FIS R, SUF R, TET R | *aadA2*, *sul1*, *fosA7.3*, *qnrB19*, *tet*(A) | SAMN14359769 |
| 16 | N19S1260 | Braenderup | 2019 | Pork chops | Swine | 32 (R) | ≤0.015 (S) | AMC R, AMP R, AXO R, CHL I, FIS R, FOX R, GEN R, SUF R, TET R | ant(2'')-Ia, aph(3'')-Ib, aph(3')-Ia, aph(6)-Id, *bla*CMY-2, *sul2*, *bla*TEM-1, *cmlA5*, *floR*, *tet*(A) | SAMN13108535 |
| 17 | N19S0536 | Reading | 2019 | Ground turkey | Turkey | ≤0.25 (S) | 1 (R) | AMC I, AMP R, CIP R, FIS R, NAL R, SUF R, TET R | aph(3'')-Ib, aph(6)-Id, *sul2*, *bla*TEM-1, *qnrB19*, *tet*(A) | SAMN12142568 |
| 18 | N19S0800 | Johannesburg | 2019 | Pork chops | Swine | ≤0.25 (S) | 1 (R) | AMP R, CHL I, CIP R, FIS R, GEN R, NAL R, SUF R, TET R | aac(3)-IVa, *aadA7*, *sul1*, *bla*TEM-1, *qnrB19*, *tet*(B) | SAMN14113278 |
| 19 | N19S2010 | Senftenberg | 2019 | Pork chops | Swine | ≤0.25 (S) | 0.5 (I) | CIP I, NAL R | *qnrB19* | SAMN13832246 |
| 20 | N21S0231 | Infantis | 2021 | Chicken | Chicken | >64 (R) | 0.125 (I) | AMP R, AXO R, CHL R, CIP I, COT R, FIS R, GEN I, NAL R, SUF R, TET R | aac(3)-IVa, *aadA1*, aph(3')-Ia, *bla*CTX-M-65, *dfrA14*, *sul1*, *floR*, *gyrA* D87Y, *tet*(A) | SAMN27521854 |
| 21 | N21S0323 | Infantis | 2021 | Chicken | Chicken | >64 (R) | 0.25 (I) | AMP R, AXO R, CHL R, CIP I, COT R, FIS R, GEN R, NAL R, SUF R, TET R | aac(3)-IVa, *aadA1*, aph(3')-Ia, *bla*CTX-M-65, *dfrA14*, *sul1*, *floR*, *gyrA* D87Y, *tet*(A) | SAMN38156171 |
| 22 | N19S1710 | Johannesburg | 2019 | Pork chops | Swine | ≤0.25 (S) | 1 (R) | AMP R, CHL I, CIP R, FIS R, GEN R, NAL R, SUF R, TET R | aac(3)-IVa, *aadA7*, *sul1*, *bla*TEM-1, *qnrB19*, *tet*(B) | SAMN13281065 |
| 23 | N19S0532 | Infantis | 2019 | Chicken | Chicken | >64 (R) | 0.25 (I) | AMP R, AXO R, CHL R, CIP I, FIS R, GEN R, NAL R, SUF R, TET R | *aadA1*, *bla*CTX-M-65, *sul1*, *bla*TEM-1, *gyrA* D87Y, *tet*(A) | SAMN12138682 |
| 24 | N19S0552 | Infantis | 2019 | Ground turkey | Turkey | >64 (R) | 0.25 (I) | AMP R, AXO R, CIP I, FIS R, NAL R, SUF R, TET R | *aadA1*, *bla*CTX-M-65, *sul1*, *bla*TEM-1, *gyrA* D87Y, *tet*(A) | SAMN12843007 |
| 25 | N19S1491 | Alachua | 2019 | Ground turkey | Turkey | 32 (R) | ≤0.015 (S) | AMC R, AMP R, AXO R, CHL R, FIS R, FOX R, SUF R, TET R | aph(3'')-Ib, aph(3')-Ia, aph(6)-Id, *bla*CMY-2, *sul2*, *fosA7.8*, *bla*TEM-1, *cmlA5*, *floR*, *tet*(A) | SAMN19372114 |
| MIC values are reported in µg/mL. S, susceptible; I, intermediate; R, resistant; MIC, minimum inhibitory concentration; AMR, antimicrobial resistance; MDR, multidrug resistance; AXO, ceftriaxone; CIP, ciprofloxacin; AMC, amoxicillin–clavulanate; AMP, ampicillin; CHL, chloramphenicol; COT, trimethoprim–sulfamethoxazole; FIS, sulfisoxazole; FOX, cefoxitin; GEN, gentamicin; NAL, nalidixic acid; SUF, sulfamethoxazole; TET, tetracycline. The non-susceptibility profile includes antimicrobials classified as intermediate or resistant. AMR determinants were identified using whole-genome sequencing data. “None detected” indicates that no phenotypic non-susceptibility was identified for the antimicrobials tested and does not imply the absence of AMR genes. | | | | | | | | | | |

**Table S2. Association between antimicrobial MICs and growth fitness parameters.** Linear regression estimates describing the associations between log₂-transformed MIC values and model-derived growth parameters for ceftriaxone and ciprofloxacin. Slope estimates (β) represent the expected change in each growth parameter associated with a two-fold increase in MIC. Results are presented as estimates with 95% confidence intervals (CIs) and P values.

| **Antibiotic** | **Growth parameter** | **log2 MIC slope β** | **95% CI** | **P value** |
| --- | --- | --- | --- | --- |
| Ceftriaxone | Intrinsic growth rate / hour | 0.0005 | -0.0056 to 0.0065 | 0.877 |
| Ceftriaxone | Maximum density | 0.0035 | -0.0072 to 0.0142 | 0.505 |
| Ceftriaxone | Lag duration | 0.0189 | -0.0476 to 0.0853 | 0.560 |
| Ciprofloxacin | Intrinsic growth rate / hour | -0.0013 | -0.0101 to 0.0075 | 0.762 |
| Ciprofloxacin | Maximum density | 0.0106 | -0.0042 to 0.0253 | 0.157 |
| Ciprofloxacin | Lag duration | -0.0399 | -0.1359 to 0.0560 | 0.396 |

**Table S3. Association between AMR gene burden and growth fitness parameters in *Salmonella enterica*.** Linear regression estimates describing the relationships between the number of unique AMR genes detected per strain and model-derived intrinsic growth rate, maximum density, and lag duration. Results are presented with 95% confidence intervals (CIs) and P values.

| **Growth parameter** | **Model term** | **Slope estimate** | **95% CI** | **P value** |
| --- | --- | --- | --- | --- |
| Intrinsic growth rate / hour | Number of unique AMR genes | 0.0028 | −0.0036 to 0.0093 | 0.373 |
| Maximum density | Number of unique AMR genes | 0.0002 | −0.0116 to 0.0121 | 0.969 |
| Lag duration | Number of unique AMR genes | 0.0229 | −0.0494 to 0.0952 | 0.516 |

**Table S4. Differences in growth fitness parameters by antimicrobial resistance-class group.** Estimated differences in model-derived growth parameters between non-multidrug-resistant (non-MDR) and multidrug-resistant (MDR) Salmonella enterica strains. MDR was defined as resistance to three or more antimicrobial classes. Estimates represent differences between resistance groups, with 95% confidence intervals (CIs) and P values reported for each growth parameter.

| **Growth parameter** | **Contrast** | **Estimate** | **95% CI** | **P value** |
| --- | --- | --- | --- | --- |
| Intrinsic growth rate / hour | Resistant 1–3 classes − MDR >3 classes | 0.0070 | −0.0312 to 0.0453 | 0.705 |
| Maximum density | Resistant 1–3 classes − MDR >3 classes | −0.0179 | −0.0950 to 0.0592 | 0.632 |
| Lag duration | Resistant 1–3 classes − MDR >3 classes | 0.1043 | −0.2825 to 0.4911 | 0.578 |
